# High-volume cataract surgery in Lahan, Nepal

**Published:** 2022-12-16

**Authors:** Reena Yadav, Abhishek Roshan, Sanjay Kumar Singh

**Affiliations:** 1 Cornea Specialist: Sagarmartha Choudhary Eye Hospital, Lahan, Nepal and Research Lead: Nepal, London School of Hygiene & Tropical Medicine and NNJS.; 2 Senior Hospital Manager: Sagarmatha Chaudhary Eye Hospital, Lahan, Nepal.; 3 Associate Professor and Medical Superintendent: Sagarmatha Chaudhary Eye Hospital, Lahan, Nepal.

**The efficient, team-based approach to cataract surgery practiced at Sagarmartha Choudhary Eye Hospital increases output and reduces outlay by ensuring that everyone’s time is used efficiently – thereby making surgery more affordable.**

**Figure:**
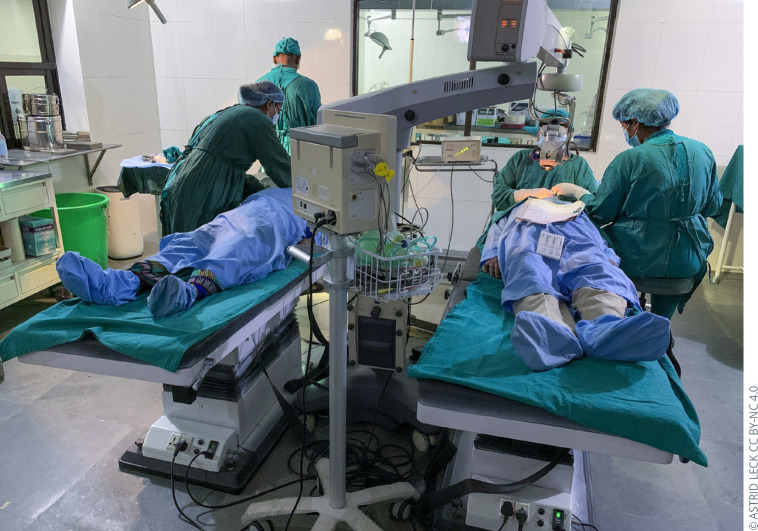
The patient on the left is being prepared for surgery while the surgeon is busy with the patient on the right. **NEPAL**

Sagarmartha Choudhary Eye Hospital (SCEH) is a 450-bed hospital in eastern Nepal, in the terai (lowlands) region near the border with India. It served more than 48,000 cataract patients in 2021: 60% from Nepal and 40% from India. During the peak season, 300–400 cataract operations take place every day, six to seven days a week. On average, surgeons perform 60–70 cataract operations per day, or up to 100 operations in a 12-hour shift during the peak season.

**“The hospital routinely monitors the outcomes of surgery in order to improve quality and improve standards, which has enabled it to develop an excellent reputation.”**

The hospital routinely monitors the outcomes of surgery in order to improve quality and improve standards, which has enabled it to develop an excellent reputation. Most patients believe the surgery offered is affordable. Manual small-incision cataract surgery costs 1,200 Nepalese rupees per eye (less than US $10), which is approximately 10% of the monthly minimum wage in both Nepal and the neighbouring Indian state of Bihar. As a result, SCEH no longer actively promotes its cataract surgery services in Nepal, because there is no perceived need to do so. In India, however, there are cataract motivators in the community who recruit patients and help them by arranging bus travel to the hospital.

## How is high output achieved?

The whole process is highly organised; every staff member, from surgeon to security guard, is clear about their role in the patient journey.

Each surgeon works between two operating tables simultaneously. By the time a surgeon has removed the first patient’s cataractous lens and tied the conjunctival suture, the next patient, on the adjacent table, is ready for their lens to be removed.

Surgeons use the ‘Fishhook’ surgical technique^1^ to deliver the nucleus, and the entire procedure takes just 3–4 minutes to perform on the fully prepared patient.

## Clinical and surgical team

The clinical team consists of two general consultant (senior) ophthalmologists and five consultant ophthalmologists who are also subspecialists: a paediatric ophthalmologist, a cornea subspecialist, a glaucoma subspecialist, and two retina subspecialists. All the subspecialists split their days between cataract surgery and their own subspecialty.

There are also seven anterior segment fellows: recently graduated ophthalmologists from Nepal who are at different stages of a rigorous 2-year in-house training programme in cataract surgery (see panel).

Ophthalmic assistants at SCEH have an extended role. They perform a detailed eye examination of each patient and take an ocular and systemic history. The level of difficulty of the operation and the likelihood of complications are then discussed with the senior supervising surgeon, who decides which patients to assign to which trainees, based on their level of experience. This approach ensures high quality and fewer complications.

SCEH also employs eye health workers (EHWs) who are trained to perform pre-operative checks and prepare patients for surgery. This includes giving the peribulbar block, applying the bridle suture (superior rectus muscle traction suture), placing the speculum, performing peritomy (opening the conjunctiva), and cauterising the highly vascular scleral tissue.

## Managing quality

Every three months, cataract operations are audited and staff present and discuss difficult/challenging cases to improve practice.

If a surgeon or trainee surgeon encounters complications, a senior surgeon will step in if needed. The surgeon responsible is asked to follow the patient’s progress closely and give a presentation that includes a discussion of the complication and how it could have been avoided and/or better managed (this can include reviewing video recordings). The trainee surgeon may then be supported with closer supervision if needed.

Cataract surgical outcome is measured on the first day after surgery, at the end of the first month after surgery, and at the three-month follow-up visit. At the one-month follow-up, more than 60% of all cataract patients have uncorrected visual acuity of 6/18 or better.

## Outlay

Ordering consumables in bulk (made possible due to the high volume of surgery) helps to keep the costs down. Most surgical instruments are sterilised and re-used, e.g., keratome and crescent blades (typically can be used for five cases), Simcoe cannulas (cleaned then steam sterilised and reused).

The greatest saving in terms of outlay is due to the efficiency with which patients move through the eye care system. The systems developed at SCEH, such as training eye health workers to prepare patients and give anesthesia, and setting up the operating theatre so one patient is being prepared while the surgeon is still operating on another patient, reduce the time the patient is in theatre, which means that everyone’s time is used more efficiently. This reduces the overhead costs per patient and therefore the overall outlay, which supports SCEH to offer surgery at an affordable price.

## Sustainability

SCEH has a separate outpatient department for patients on higher incomes and offers a range of eye services, including phacoemulsification cataract surgery. Income from this department subsidises low-income patients. SCEH also benefits from donor agencies who support the costs of equipment, human resource development, and surgical consumables.

**Figure:**
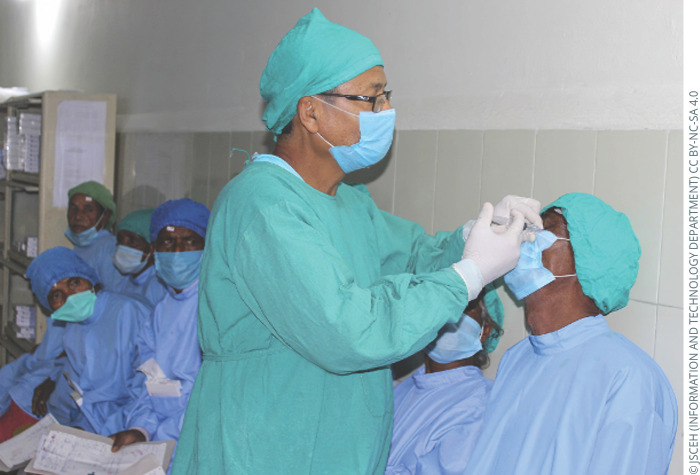
An eye health worker (EHW) giving a peribulbar block a few minutes before taking the patient to the operating table. A sterile eye pad is kept over the eye and the patient is asked to apply gentle pressure with the palm of his hand. Eye movement and pressure is checked before sending the patient for surgery. **NEPAL**

## Supporting women

Even though women and girls in Nepal have a greater burden of blindness than men and boys, they are less likely to visit eye hospitals, for a variety of reasons.^2^ SCEH monitors uptake of cataract services separately for male and female patients, and has put in place measures to make the facilities female friendly, for example by offering separate registration counters, queues and toilets for men and women, an enclosed breastfeeding space, and a female counsellor for female patients. At present, around 3% more cataract operations are performed in women than in men.

*The authors would like to thank Astrid Leck and Elmien Wolvaardt for their contributions to this article.*

**Cataract surgery training**

Cataract surgery trainees, known as anterior segment fellows, undergo a rigorous two-year training programme.

Candidates must be ophthalmologists registered with Nepal’s Medical Council and undergo a written exam and interview at SCEH before being considered for the programme. The successful candidates must also pass the SCEH protocol exam before being eligible to examine patients in the outpatient department (OPD).

Surgical training starts after one month of OPD exposure. Training begins with two days of observing surgery in the operating theatre, followed by a week of suturing practice in the wet lab. After that, the trainee surgeons perform skin suturing, under supervision, in adult dacrocystorhinostomy patients. Once confident in skin suturing, they are given opportunities to perform supervised corneal suturing in adult patients with corneal or corneoscleral injury who have minimal visual potential.

Once hand-eye coordination is well established, and fellows are comfortable handling ocular injury surgery independently, they are given selected cataract surgery cases.

Cataract surgery training is started systematically. Fellows are trained in each step under supervision, for a period of one month. After evaluation by the supervisor/mentor, they are eligible to perform independent cataract surgery. Complications are managed by the supervisor/ mentor initially, and complication management is gradually handed over to the trainees, depending upon their individual performance.

For more information, visit **www.erec-p.org/sagarmatha**
